# Analyzing Public Google Search Interest in Measles Within Canada: Identifying Key Moments for Targeted Risk Communication

**DOI:** 10.2196/75025

**Published:** 2025-07-09

**Authors:** Mohammad Jokar, Diego Nobrega

**Affiliations:** 1 Faculty of Veterinary Medicine, University of Calgary, 3280 Hospital Drive NW, Calgary, AB, T2N 4Z6, Canada, 1 403-220-7020

**Keywords:** measles, Google search, risk communication, Canada, public search interest

## Abstract

We analyzed Google Trends data on measles-related searches in Canada from January 1 to May 21, 2025; web, news, and YouTube search trends increased significantly across provinces (all *P* values were <.05), aligning with rising case numbers. Our findings emphasize the importance of timely, targeted risk communication for enhancing public awareness and responses during this outbreak.

## Introduction

Measles is a contagious, airborne, viral infection that can lead to serious complications, particularly among infants, older adults, and individuals who are immunocompromised [1]. Canada saw a sharp rise in cases—from 12 in 2023 to 2429 by May 2025—that was mostly linked to international travel [2]. Despite a highly effective vaccine, immunization rates remain below the 95% herd immunity threshold, with regional disparities [1,3]. Infodemiology helps with tracking public health information trends, identifying misinformation, and guiding risk communication [4,5]. As measles cases rise, monitoring public search interest (PSI) is crucial for improving awareness and ensuring the effective dissemination of trustworthy health information.

## Methods

This study used Google Trends (GT) to analyze PSI in measles from January 1 to May 21, 2025, across web, news, and YouTube searches on Google in Canada, using the disease topic “Measles” (Multimedia Appendix 1) [6-8]. Relative search volumes (RSVs), as provided by GT, were compared with confirmed measles case data in Canada, and significant changes over time were assessed by using statistical tests in R (v4.4.3; R Foundation for Statistical Computing; Multimedia Appendix 1).

## Results

**Figure 1.**
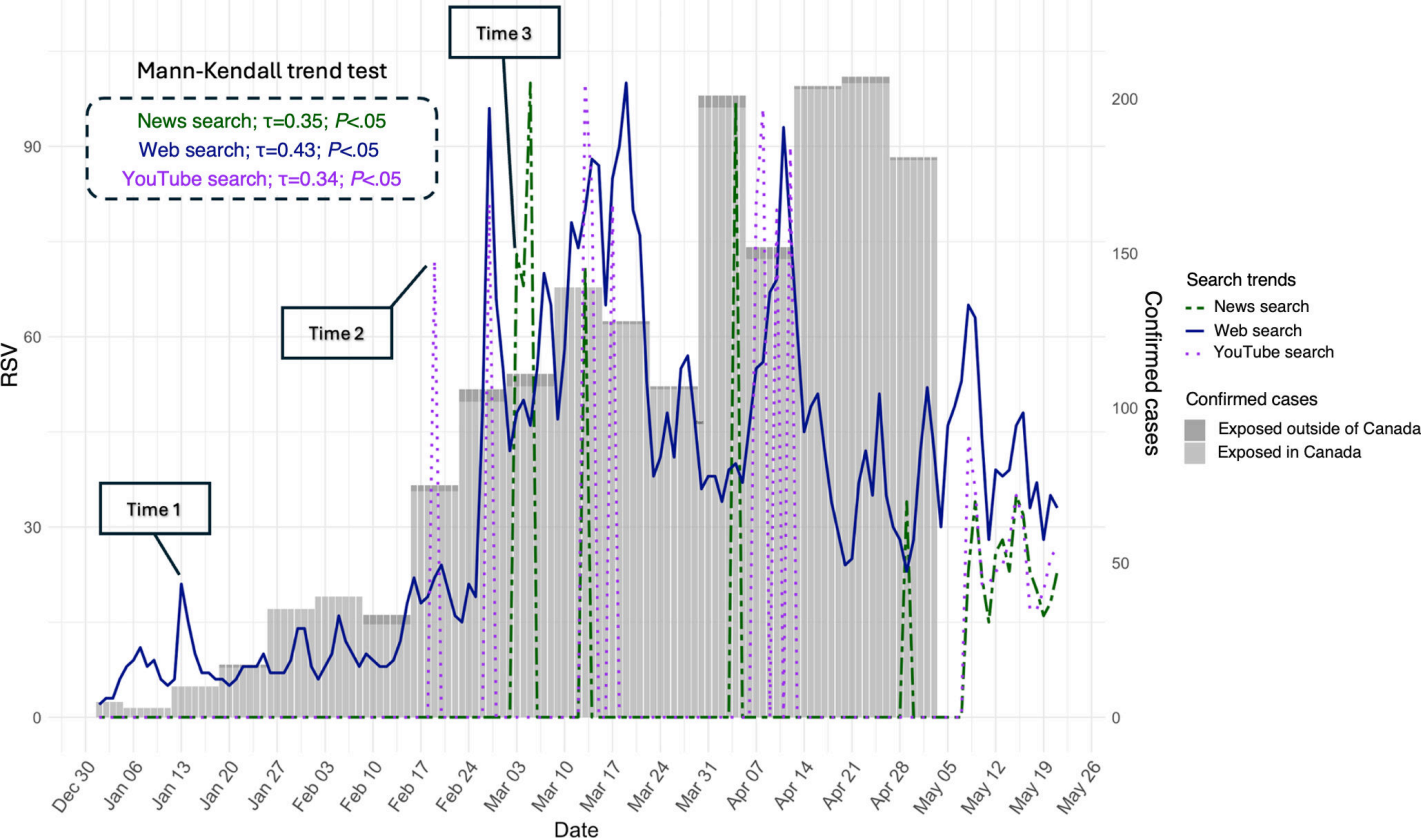
Google search trends for “Measles” in Canada from January 1 to May 21, 2025, highlighting key time points for targeted risk communication (January 13, February 19, March 3), along with the Mann-Kendall trend test results. RSV: relative search volume.

During the study period, PSI in measles mildly increased on January 13, when 10 confirmed cases were reported, which might be the ideal time to enhance targeted risk communication. The findings demonstrate that Google web search was the primary method people used to seek measles-related information before the sharp increase to 35 cases in the last week of January. The trend for web searches then significantly rose on February 27, coinciding with 102 confirmed cases. There was also an increase in YouTube searches on February 19, coinciding with 75 confirmed cases and making this another key moment for strengthening risk communication through YouTube content. On March 3 in Canada, there was a noticeable increase in searching for news content on Google, when 111 confirmed cases were reported. This can be considered another optimal time for circulating reliable information by focusing on news content (Figure 1, Tables S1 and S2 in Multimedia Appendix 1). The Mann-Kendall trend test revealed significant positive trends (all *P* values were <.05) for web, news, and YouTube searches (Figure 1).

**Figure 2.**
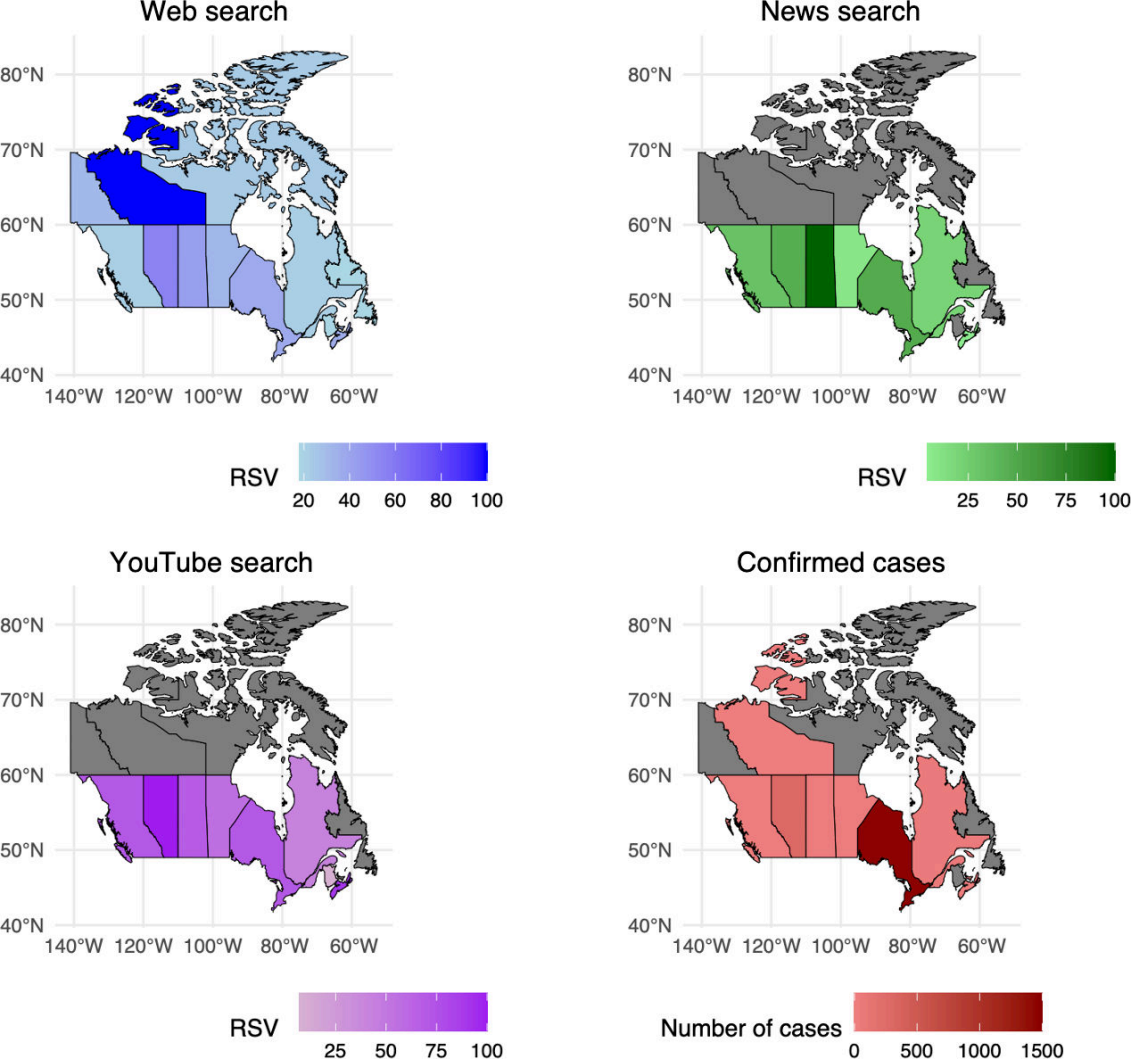
RSVs for the topic “Measles” across different search types on Google in various provinces of Canada (January 1 to May 21, 2025), along with a choropleth map of confirmed cases within Canada in 2025. RSV: relative search volume.

People in Ontario and Alberta exhibited high interest in YouTube and moderate interest in Google web searches. These provinces also reported the highest numbers of confirmed measles cases—1460 in Ontario and 287 in Alberta. Similarly, people in Quebec showed moderate interest in YouTube searches, corresponding to 36 confirmed cases. People in Saskatchewan also showed high interest in news searches and moderate interest in YouTube searches, corresponding to 27 confirmed cases. People in British Columbia and Manitoba showed moderate interest in YouTube searches, corresponding to 8 and 24 confirmed cases, respectively. In Nova Scotia and the Northwest Territories, people showed high interest in YouTube and web searches, respectively, even though only 1 confirmed case was reported in each location (Figure 2, Table S3 in Multimedia Appendix 1).

## Discussion

This study identified 3 time points when PSI increased across web, news, and YouTube searches on Google. Such moments present opportunities for health authorities to strategically deliver accurate information and reduce misinformation [4,6]. Moreover, in Ontario and Alberta, where the highest numbers of cases were reported, interest in searching on YouTube was high, reflecting heightened demand for visual content. The rising measles cases in Canada and increased PSI underscore the need for timely, region-specific risk communication, which is consistent with past findings linking high search interest in diseases like mpox (monkeypox) and COVID-19 to local incidence rates [4,9]. YouTube is an important platform that health authorities use to disseminate crucial information (eg, symptom explanations and vaccine safety information) during crises (eg, the COVID-19 pandemic). This is supported by research indicating that visuals significantly improve comprehension and retention of health messages, particularly benefiting populations with lower literacy by making complex information more accessible and understandable and by positively influencing health behaviors [10]. Another positive aspect of YouTube is 2-way engagement, as it provides a comment section where health authorities can consider strategies for improving one-on-one communication and direct responses [10]. It is important to note that GT only provides RSV data; it does not provide the absolute number of searches. Therefore, developing local datasets would be helpful for better PSI tracking during measles outbreaks in Canada. Search surges across the web, news, and YouTube offer key opportunities for region-specific public health messaging. Integrating real-time search monitoring into outbreak communication plans, especially on YouTube, can enhance the delivery of accurate, timely information.

## Supplementary material

10.2196/75025 Multimedia Appendix 1 Supplementary materials regarding data collection, data analysis, daily relative search volume, number of confirmed measles cases, and the R code used for analyses.

## Abbreviations

GT: Google Trends
mpox: monkeypox
PSI: public search interest
RSV: relative search volume
