# Supplementary material for: Analyzing Public Google Search Interest in Measles Within Canada: Identifying Key Moments for Targeted Risk Communication
Source: J Med Internet Res. 2025 Jul 9;27:e75025. doi: 10.2196/75025 (PMC12266579; doi:10.2196/75025)
Supplement: Multimedia Appendix 1 [file jmir-v27-e75025-s001.docx]

**Appendix**

**Methods**

**Data Collection**

To assess public search interest (PSI) related to measles, we utilized Google Trends (GT), a publicly available tool (https://trends.google.com/) that measures the popularity of search queries over time using Relative Search Volume (RSV). RSV values range from 0 to 100, representing the proportion of searches for a given topic or search query relative to all searches, with higher values indicating increased (PSI) in that topic or query [6].

We conducted a topic-based search for “Measles,” by choosing “Measles” as “Disease topic” which aggregates RSV data from semantically related terms across different languages [6–8]. We retrieved data across three GT search types: Web Search, News Search, and YouTube Search. Searches were not restricted by content category (i.e., “All Categories” was selected), and data were collected for the period from January 1 to May 21, 2025. To contextualize RSV patterns with epidemiological data, we obtained weekly confirmed measles case counts in Canada from the Weekly surveillance reports and monitoring maps for measles and rubella provided by the Public Health Agency of Canada (https://health-infobase.canada.ca/measles-rubella/) [2].

**Data Analysis**

We analyzed trends in RSV to determine whether PSI changed significantly during the observation period. A Mann-Kendall trend test was employed to detect monotonic trends in RSV over time, which is a non-parametric test used for identifying trends in epidemiological time series data. Analyses were conducted separately for each Google Trends search type (Web, News, and YouTube). Statistical analysis and data visualizations were performed using R version 4.4.3 (code available in this appendix) with a significance level of 0.05, and the raw data used are presented in Tables S1 to S3.

| **Table S1.** Daily relative search volume (RSV) for “Measles” (disease as topic) based on Google web, news, and YouTube searches in Canada, January 1 to May 21, 2025. | | | |
| --- | --- | --- | --- |
| **Day** | **Web Search** | **News Search** | **YouTube Search** |
| 2025-01-01 | 2 | 0 | 0 |
| 2025-01-02 | 3 | 0 | 0 |
| 2025-01-03 | 3 | 0 | 0 |
| 2025-01-04 | 6 | 0 | 0 |
| 2025-01-05 | 8 | 0 | 0 |
| 2025-01-06 | 9 | 0 | 0 |
| 2025-01-07 | 11 | 0 | 0 |
| 2025-01-08 | 8 | 0 | 0 |
| 2025-01-09 | 9 | 0 | 0 |
| 2025-01-10 | 6 | 0 | 0 |
| 2025-01-11 | 5 | 0 | 0 |
| 2025-01-12 | 6 | 0 | 0 |
| 2025-01-13 | 21 | 0 | 0 |
| 2025-01-14 | 15 | 0 | 0 |
| 2025-01-15 | 10 | 0 | 0 |
| 2025-01-16 | 7 | 0 | 0 |
| 2025-01-17 | 7 | 0 | 0 |
| 2025-01-18 | 6 | 0 | 0 |
| 2025-01-19 | 6 | 0 | 0 |
| 2025-01-20 | 5 | 0 | 0 |
| 2025-01-21 | 6 | 0 | 0 |
| 2025-01-22 | 8 | 0 | 0 |
| 2025-01-23 | 8 | 0 | 0 |
| 2025-01-24 | 8 | 0 | 0 |
| 2025-01-25 | 10 | 0 | 0 |
| 2025-01-26 | 7 | 0 | 0 |
| 2025-01-27 | 7 | 0 | 0 |
| 2025-01-28 | 7 | 0 | 0 |
| 2025-01-29 | 9 | 0 | 0 |
| 2025-01-30 | 14 | 0 | 0 |
| 2025-01-31 | 14 | 0 | 0 |
| 2025-02-01 | 8 | 0 | 0 |
| 2025-02-02 | 6 | 0 | 0 |
| 2025-02-03 | 8 | 0 | 0 |
| 2025-02-04 | 10 | 0 | 0 |

| **Table S1.** Continued | | | |
| --- | --- | --- | --- |
| **Day** | **Web Search** | **News Search** | **YouTube Search** |
| 2025-02-05 | 16 | 0 | 0 |
| 2025-02-06 | 12 | 0 | 0 |
| 2025-02-07 | 10 | 0 | 0 |
| 2025-02-08 | 8 | 0 | 0 |
| 2025-02-09 | 10 | 0 | 0 |
| 2025-02-10 | 9 | 0 | 0 |
| 2025-02-11 | 8 | 0 | 0 |
| 2025-02-12 | 8 | 0 | 0 |
| 2025-02-13 | 9 | 0 | 0 |
| 2025-02-14 | 12 | 0 | 0 |
| 2025-02-15 | 18 | 0 | 0 |
| 2025-02-16 | 22 | 0 | 0 |
| 2025-02-17 | 18 | 0 | 0 |
| 2025-02-18 | 19 | 0 | 0 |
| 2025-02-19 | 22 | 0 | 72 |
| 2025-02-20 | 24 | 0 | 0 |
| 2025-02-21 | 20 | 0 | 0 |
| 2025-02-22 | 16 | 0 | 0 |
| 2025-02-23 | 15 | 0 | 0 |
| 2025-02-24 | 21 | 0 | 0 |
| 2025-02-25 | 19 | 0 | 0 |
| 2025-02-26 | 48 | 0 | 0 |
| 2025-02-27 | 96 | 0 | 81 |
| 2025-02-28 | 66 | 0 | 0 |
| 2025-03-01 | 55 | 0 | 0 |
| 2025-03-02 | 42 | 0 | 0 |
| 2025-03-03 | 48 | 73 | 0 |
| 2025-03-04 | 50 | 68 | 0 |
| 2025-03-05 | 46 | 100 | 0 |
| 2025-03-06 | 55 | 0 | 0 |
| 2025-03-07 | 70 | 0 | 0 |
| 2025-03-08 | 65 | 0 | 0 |
| 2025-03-09 | 47 | 0 | 0 |
| 2025-03-10 | 58 | 0 | 0 |
| 2025-03-11 | 78 | 0 | 0 |
| 2025-03-12 | 74 | 0 | 0 |
| 2025-03-13 | 80 | 71 | 100 |

| **Table S1.** Continued | | | |
| --- | --- | --- | --- |
| **Day** | **Web Search** | **News Search** | **YouTube Search** |
| 2025-03-14 | 88 | 0 | 83 |
| 2025-03-15 | 87 | 0 | 0 |
| 2025-03-16 | 65 | 0 | 0 |
| 2025-03-17 | 85 | 0 | 81 |
| 2025-03-18 | 90 | 0 | 0 |
| 2025-03-19 | 100 | 0 | 0 |
| 2025-03-20 | 80 | 0 | 0 |
| 2025-03-21 | 76 | 0 | 0 |
| 2025-03-22 | 53 | 0 | 0 |
| 2025-03-23 | 38 | 0 | 0 |
| 2025-03-24 | 41 | 0 | 0 |
| 2025-03-25 | 48 | 0 | 0 |
| 2025-03-26 | 41 | 0 | 0 |
| 2025-03-27 | 55 | 0 | 0 |
| 2025-03-28 | 57 | 0 | 0 |
| 2025-03-29 | 47 | 0 | 0 |
| 2025-03-30 | 36 | 0 | 0 |
| 2025-03-31 | 38 | 0 | 0 |
| 2025-04-01 | 38 | 0 | 0 |
| 2025-04-02 | 34 | 0 | 0 |
| 2025-04-03 | 39 | 0 | 0 |
| 2025-04-04 | 40 | 97 | 0 |
| 2025-04-05 | 37 | 0 | 0 |
| 2025-04-06 | 46 | 0 | 0 |
| 2025-04-07 | 55 | 0 | 81 |
| 2025-04-08 | 56 | 0 | 96 |
| 2025-04-09 | 67 | 0 | 0 |
| 2025-04-10 | 69 | 0 | 80 |
| 2025-04-11 | 93 | 0 | 0 |
| 2025-04-12 | 77 | 0 | 90 |
| 2025-04-13 | 61 | 0 | 0 |
| 2025-04-14 | 45 | 0 | 0 |
| 2025-04-15 | 49 | 0 | 0 |
| 2025-04-16 | 51 | 0 | 0 |
| 2025-04-17 | 42 | 0 | 0 |
| 2025-04-18 | 34 | 0 | 0 |
| 2025-04-19 | 29 | 0 | 0 |

| **Table S1.** Continued | | | |
| --- | --- | --- | --- |
| **Day** | **Web Search** | **News Search** | **YouTube Search** |
| 2025-04-20 | 24 | 0 | 0 |
| 2025-04-21 | 25 | 0 | 0 |
| 2025-04-22 | 37 | 0 | 0 |
| 2025-04-23 | 42 | 0 | 0 |
| 2025-04-24 | 35 | 0 | 0 |
| 2025-04-25 | 51 | 0 | 0 |
| 2025-04-26 | 35 | 0 | 0 |
| 2025-04-27 | 30 | 0 | 0 |
| 2025-04-28 | 28 | 0 | 0 |
| 2025-04-29 | 23 | 34 | 0 |
| 2025-04-30 | 28 | 0 | 0 |
| 2025-05-01 | 42 | 0 | 0 |
| 2025-05-02 | 52 | 0 | 0 |
| 2025-05-03 | 42 | 0 | 0 |
| 2025-05-04 | 30 | 0 | 0 |
| 2025-05-05 | 46 | 0 | 0 |
| 2025-05-06 | 49 | 0 | 0 |
| 2025-05-07 | 53 | 0 | 0 |
| 2025-05-08 | 65 | 23 | 44 |
| 2025-05-09 | 63 | 34 | 36 |
| 2025-05-10 | 44 | 22 | 21 |
| 2025-05-11 | 28 | 15 | 21 |
| 2025-05-12 | 39 | 26 | 23 |
| 2025-05-13 | 38 | 28 | 24 |
| 2025-05-14 | 39 | 23 | 28 |
| 2025-05-15 | 46 | 35 | 35 |
| 2025-05-16 | 48 | 32 | 31 |
| 2025-05-17 | 33 | 23 | 17 |
| 2025-05-18 | 37 | 20 | 17 |
| 2025-05-19 | 28 | 16 | 20 |
| 2025-05-20 | 35 | 18 | 25 |
| 2025-05-21 | 33 | 23 | 27 |
| *Source: Google Trends, searching “Measles” as a disease topic (https://trends.google.com/). | | | |

| **Table S2.** Number of confirmed measles cases by exposure source and epidemiological week in Canada, 2025. | | |
| --- | --- | --- |
| **Date Range (Epidemiological week)** | **Exposed Outside of Canada** | **Exposed in Canada** |
| Dec 29, 2024 – Jan 4, 2025 (Week 1) | 0 | 5 |
| Jan 5 – Jan 11, 2025 (Week 2) | 0 | 3 |
| Jan 12 – Jan 18, 2025 (Week 3) | 0 | 10 |
| Jan 19 – Jan 25, 2025 (Week 4) | 1 | 16 |
| Jan 26 – Feb 1, 2025 (Week 5) | 0 | 35 |
| Feb 2 – Feb 8, 2025 (Week 6) | 0 | 39 |
| Feb 9 – Feb 15, 2025 (Week 7) | 3 | 30 |
| Feb 16 – Feb 22, 2025 (Week 8) | 2 | 73 |
| Feb 23 – Mar 1, 2025 (Week 9) | 4 | 102 |
| Mar 2 – Mar 8, 2025 (Week 10) | 4 | 107 |
| Mar 9 – Mar 15, 2025 (Week 11) | 0 | 139 |
| Mar 16 – Mar 22, 2025 (Week 12) | 1 | 127 |
| Mar 23 – Mar 29, 2025 (Week 13) | 1 | 106 |
| Mar 30 – Apr 5, 2025 (Week 14) | 4 | 197 |
| Apr 6 – Apr 12, 2025 (Week 15) | 4 | 148 |
| Apr 13 – Apr 19, 2025 (Week 16) | 1 | 203 |
| Apr 20 – Apr 26, 2025 (Week 17) | 2 | 205 |
| Apr 27 – May 3, 2025 (Week 18) | 1 | 180 |
| * Source: Measles and rubella weekly monitoring report (https://health-infobase.canada.ca/) | | |

| **Table S3.** Relative search volume (RSV) for “Measles” (disease as topic) across Google web, news, and YouTube searches, and confirmed measles case counts in different regions of Canada from January 1 to May 21, 2025. | | | | |
| --- | --- | --- | --- | --- |
| **Region** | **Web Search** | **News Search** | **YouTube Search** | **Confirmed cases** |
| Alberta | 55 | 45 | 100 | 287 |
| British Columbia | 21 | 32 | 71 | 8 |
| Manitoba | 32 | 8 | 56 | 24 |
| New Brunswick | 18 | - | 7 | 0 |
| Newfoundland and Labrador | 18 | - | - | 0 |
| Northwest Territories | 100 | - | - | 1 |
| Nova Scotia | 36 | 4 | 90 | 1 |
| Nunavut | 23 | - | - | 0 |
| Ontario | 37 | 47 | 71 | 1460 |
| Prince Edward Island | 30 | - | - | 2 |
| Quebec | 21 | 20 | 42 | 36 |
| Saskatchewan | 43 | 100 | 69 | 27 |
| Yukon Territory | 31 | - | - | 0 |
| Sources: Google Trends, searching “Measles” as a disease topic (https://trends.google.com/); Measles and Rubella Weekly Monitoring Report (https://health-infobase.canada.ca/). | | | | |

**R Code for Analyzing and Visualizing Google Search Trends and Confirmed Case Data Using Mann-Kendall Trend Tests**

# Load required packages

library(ggplot2)

library(dplyr)

library(tidyr)

library(readr)

library(Kendall)

# Load the dataset

data <- read_csv("path/to/your/search_trends.csv")

# Convert 'Day' column to Date format

data$Day <- as.Date(data$Day)

# Filter data for the year 2025

data_2025 <- data %>%

filter(format(Day, "%Y") == "2025")

# Reshape search trends to long format

data_long <- data_2025 %>%

pivot_longer(cols = c(`Web Search`, `News Search`, `YouTube Search`),

names_to = "Search_Type",

values_to = "Search_Volume")

# Reshape confirmed cases by exposure type

data_reshaped <- data_2025 %>%

pivot_longer(cols = c(`Confirmed cases (exposed outside of Canada)`,

`Confirmed cases (exposed in Canada)`),

names_to = "Exposure_Type",

values_to = "Confirmed_Cases") %>%

mutate(Exposure_Type = factor(Exposure_Type,

levels = c("Confirmed cases (exposed outside of Canada)",

"Confirmed cases (exposed in Canada)")))

# Perform Mann-Kendall trend tests

result_web_search <- MannKendall(data_2025$`Web Search`)

result_news_search <- MannKendall(data_2025$`News Search`)

result_youtube_search <- MannKendall(data_2025$`YouTube Search`)

# Extract Tau and p-values

extract_results <- function(result) {

list(tau = result$tau, p = result$sl)

}

res_web <- extract_results(result_web_search)

res_news <- extract_results(result_news_search)

res_youtube <- extract_results(result_youtube_search)

# Function to format p-values for display

format_p_value <- function(p) {

if (p < 0.05) {

return("p < 0.05")

} else {

return(paste("p =", round(p, 3)))

}

}

# Annotated trend text for each search type

text_web <- paste("Web Search: Tau =", round(res_web$tau, 2), ",", format_p_value(res_web$p))

text_news <- paste("News Search: Tau =", round(res_news$tau, 2), ",", format_p_value(res_news$p))

text_youtube <- paste("YouTube Search: Tau =", round(res_youtube$tau, 2), ",", format_p_value(res_youtube$p))

# Create the combined plot

p <- ggplot() +

geom_bar(data = data_reshaped,

aes(x = Day, y = Confirmed_Cases, fill = Exposure_Type),

stat = "identity", position = "stack", alpha = 0.7) +

geom_line(data = data_long,

aes(x = Day, y = Search_Volume, color = Search_Type, linetype = Search_Type),

size = 1) +

scale_y_continuous(name = "Relative Search Volume (RSV)") +

labs(x = "Date", fill = "Exposure Type", color = "Search Type", linetype = "Search Type") +

theme_minimal()

# Optional: print annotations (can be added to the plot if needed)

print(text_web)

print(text_news)

print(text_youtube)

# Display the plot

print(p)

**R Code for Generating Static Choropleth Maps**

# Load required libraries

library(ggplot2)

library(sf)

library(dplyr)

library(gridExtra)

library(leaflet)

library(readxl)

library(geojsonio)

# Load and clean Excel data

data <- read_excel("path/to/your/map.xlsx") |>

mutate(Region = as.character(Region),

Region = gsub("Qu√©bec", "Quebec", Region),

Region = gsub("Yukon Territory", "Yukon", Region))

# Load and prepare spatial data

canada_sf <- geojson_read("Desktop/georef-canada-province@public.geojson", what = "sp") |>

st_as_sf() |>

mutate(prov_name_en = as.character(prov_name_en))

# Merge spatial and tabular data

merged_data <- merge(canada_sf, data, by.x = "prov_name_en", by.y = "Region", all.x = TRUE)

# Function to create choropleth map

create_map <- function(column, title, colors, legend = "RSV", limits = NULL) {

ggplot(merged_data) +

geom_sf(aes(fill = .data[[column]]), color = "black", size = 0.1) +

scale_fill_gradientn(colours = colors, limits = limits) +

theme_minimal() +

labs(title = title, fill = legend) +

theme(legend.position = "bottom", plot.title = element_text(hjust = 0.5))

}

# Create individual maps

maps <- list(

create_map("Web Search", "Web Search", c("lightblue", "blue")),

create_map("News Search", "News Search", c("lightgreen", "darkgreen")),

create_map("YouTube Search", "YouTube Search", c("#D8B3D3", "purple")),

create_map("Confirmed cases", "Confirmed Cases", c("lightcoral", "darkred"),

legend = "Number of Cases", limits = c(0, 1500))

)

# Export all maps in a 2x2 layout to PDF

pdf("map_grid_output.pdf", width = 8, height = 7)

grid.arrange(grobs = maps, ncol = 2)

dev.off()
